# Collagen Partition in Polymeric Aqueous Two-Phase Systems for Tissue Engineering

**DOI:** 10.3389/fchem.2018.00379

**Published:** 2018-09-04

**Authors:** Sunil Singh, Hossein Tavana

**Affiliations:** Department of Biomedical Engineering, The University of Akron, Akron, OH, United States

**Keywords:** collagen, partition, ATPS, spheroid, 3D culture

## Abstract

Aqueous two-phase systems (ATPS) of polyethylene glycol (PEG) and dextran (DEX) are commonly used to partition proteins. Protein partition in ATPS is a complex phenomenon and depends on factors including molecular weight of polymers, and electrochemical and ionic properties of the phases. We studied the effect of polymer molecular weight on the partition of a natural protein, collagen, in several ATPS formulations made with non-ionic polymers polyethylene glycol (PEG) and dextran (DEX). We found that partitioning of collagen to an aqueous phase significantly increases when the molecular weight of the corresponding phase polymer decreases. Additionally, a large difference between the molecular weight of the phase-forming polymers was necessary to cause a significant uneven collagen distribution between the aqueous phases. We then employed one of the systems to create a three-dimensional breast cancer microtissue by entrapping a spheroid of breast cancer cells within the partitioned collagen. This convenient technique to generate 3D microtissues offers a convenient and promising approach for tissue engineering applications.

## Introduction

Aqueous two-phase systems (ATPS) may be formed by mixing aqueous solutions of two chemically incompatible polymers (Albertsson, 1961). Polyethylene glycol (PEG) and dextran (DEX) are the most commonly used polymers to form ATPS. Each ATPS has a characteristic phase diagram with a binodal curve that prescribes pairs of concentrations of polymers to result in two immiscible phases. Only polymer concentrations above the curve give a two-phase system. Separation of two distinct aqueous layers by an interface is visible and becomes more distinct by increase in the interfacial tension between these two phases (Atefi et al., 2014, 2015). ATPS are used for separation of biomolecules such as cells (SooHoo and Walker, 2009), proteins (Johansson, 1970), and nucleic acids and organelles (Walter and Larsson, 1994). High water content and low interfacial tensions of ATPS are key properties to provide a mild environment for sensitive biomolecules.

Partitioning of proteins to either phase of an ATPS is a complex process (Schmidt et al., 1994). Proteins may favor one of the phases of an ATPS or partition toward the interface. Distribution of protein molecules may be manipulated by altering the molecular weight of the polymers, concentration of polymers, ionic strength of the aqueous solutions, pH, and hydrophobicity of the polymers. For example, amylase partitioning to the top phase improved by adding salt to the aqueous solutions (Li et al., 2002). Using charged PEG increased partitioning of penicillin acylase from E. coli to the top phase in an ATPS (Gavasane and Gaikar, 2003). It was recently shown that collagen partitions to the interface of aqueous PEG and DEX phases and this was used to generate cell-containing collagen microdrops to mimic matrix contraction (Moraes et al., 2013).

Our goal was to localize collagen to one phase, rather than to the interface, to conveniently form collagen microgels in ATPS. To demonstrate the feasibility of partitioning of collagen to the bottom DEX phase, we conducted experiments with two-phase systems of different molecular weights of PEG and DEX. Then, we selected a system that favors partition of collagen to the DEX phase and entrapped a spheroid of breast cancer cells in the partitioned collagen in the DEX phase. The resulting microtissue morphologically resembles solid breast tumors where a mass of cancer cells resides in a protein matrix (Ham et al., 2016b). This novel approach will enable future studies in tumor biology and antitumor drug discovery.

## Materials and methods

### Preparation of aqueous two-phase systems

Several ATPS were prepared with polyethylene glycol (PEG) (Sigma) and dextran (DEX) (Pharmacosmos). Two different molecular weights of PEG (8kDa & 35kDa) and DEX (40kDa & 500kDa) were used to prepare four ATPS: PEG8k-DEX40k (system A), PEG8k-DEX500k (system B), PEG35k-DEX40k (system C), and PEG35k-DEX500k (system D). From each system, different stock concentrations of PEG and DEX solutions were used to form three sets of two-phase systems: 15% PEG-21% DEX, 18% PEG-24% DEX, and 24% PEG-32% DEX. All concentrations of aqueous PEG and DEX solutions were calculated in %(w/v). Polymers were dissolved in a complete growth medium. To facilitate complete dissolution of polymers, the solutions were kept in a 37°C water bath for four hours and mixed using a vortex for two min every 30 min. All PEG and DEX solutions were filtered through syringe filters of 0.2 μm pore size to remove small particles and impurities. Resulting polymer solutions were stored at 4°C.

### Construction of binodal curves

Binodal curves for systems A, B, C, and D were constructed using a titration method (Albertsson and Tjerneld, 1994). Various ATPS were prepared in a complete growth medium in 1.5 mL microcentrifuge tubes from stock PEG and DEX solutions. Medium was added to each ATPS in 5 μL increments until the interface between the top and bottom phases disappeared. Concentrations of the polymers prior to formation of one phase were determined and used to construct a binodal curve.

### Cell culture

BT474 breast cancer cells were obtained from ATCC and cultured in RPMI 1640 medium (Sigma) supplemented with 10% fetal bovine serum (FBS) (Sigma), 1% antibiotic (Thermo Fisher Scientific), 0.1 μM sodium pyruvate (Thermo Fisher Scientific), 1 μM nonessential amino acids (Thermo Fisher Scientific), and 0.1 mM Hepes buffer (Thermo Fisher Scientific). Cells were cultured in a humidified incubator at 37°C and 5% CO_2_ in a T75 flask (Thermo Fisher Scientific). BT474 cells grew in multilayer patches. Cells were rinsed with phosphate buffered saline (PBS) (Sigma) and detached using 0.25% trypsin (Thermo Fisher Scientific) for cell seeding and passaging. Cells were sub-cultured at a ratio of 1:3.

### Preparation of collagen solution

A stock solution of type I rat tail collagen (Corning) with a concentration of 8.56 mg/ml dissolved in 0.02N acetic acid was diluted to desired concentrations using the manufacturer's protocol. For example, 1 mL of 4 mg/ml collagen solution was prepared by mixing 100 μL 10X DMEM medium, 422 μL sterile distill water, 467 μL collagen stock solution, and 11 μL 1N NaOH solution. All the reagents were kept on ice during collagen preparation to maintain the temperature at 4°C and prevent premature gelation of collagen. The pH of the solution was measured by a pH meter (Mettler Toledo) and maintained at 7.5. Prepared collagen solutions were stored at 4°C for a maximum of 1 h before use.

### Partition of collagen in ATPS

Collagen partition experiments were performed with systems A-D in serum-free RPMI. From each system, three combinations were used: 15% PEG-21% DEX, 18% PEG-24% DEX, and 24% PEG-32% DEX. Equal volumes (200 μL) of a PEG solution, a DEX solution, and a 2 mg/ml type I collagen solution were mixed in a 1.5 mL microcentrifuge tube. The mixture was equilibrated at 4°C for 60 min to allow collagen to partition between the two phases and a clear interface form. Because collagen partition experiments were conducted with equal volumes of PEG, DEX, and collagen solutions, the resulting concentrations of PEG and DEX in each system in the partition assay reduced to 5% PEG-7% DEX, 6% PEG-8% DEX, and 8% PEG-10.6% DEX. Four replicates were used for each system. From each tube, the top phase solution was pipetted out first followed by the bottom phase solution. Samples were stored in separate microcentrifuge tubes.

### Hydroxyproline assay

Collagen concentration in the bottom phase of each system was quantified using a hydroxyproline assay (Sigma). Briefly, equal volumes of the sample and concentrated hydrochloric acid (~12 M HCl) were mixed in a Teflon-capped glass vial (Taylor Scientific). Next, the solution was hydrolyzed at 110°C in a hot air oven (Binder) for 16 h. Then, the vial was cooled down to room temperature and the hydrolyzed solution was transferred into a 1.5 mL centrifuge tube. The tube was centrifuged at 180 rcf for 10 min. Next, 10 μL of the supernatant was transferred into a flat-bottom 96-well plate. The plate was incubated in a 60°C oven with the lid on for 2 h for complete drying of the sample. Each sample was spiked with 0.4 μg of hydroxyproline standard to remove absorbance interference from endogenous compounds. Hydroxyproline standards were also run simultaneously to obtain a standard curve. To each well, 6 μL of chloramine T concentrate and 94 μL of an oxidation buffer was added. The plate was incubated at room temperature for 5 min. Then, 50 μL of 4-(dimethylamino)benzaldehyde concentrate and 50 μL of perchloric acid were added. The plate was incubated at 60°C for 90 min and absorbance was measured at 560 nm using a plate reader (Synergy H1M) (Biotek Instruments). The hydroxyproline standard curve was used to determine the hydroxyproline amino acid content in the sample. The collagen content was approximated by multiplying the resulting value by a factor of 7.69 (Neuman and Logan, 1950).

### Cancer cell spheroid formation

BT474 cancer cell spheroids were formed using ATPS technology (Atefi et al., 2014). PEG35k and DEX500k were used to form the spheroids. Aqueous PEG phase solution of 6.6% (w/v) and DEX phase solution of 3.2% (w/v) were prepared separately in a complete growth medium (Atefi et al., 2014). BT474 cells were mixed thoroughly with the DEX phase solution to form a cell suspension with a density of 50 × 10^3^ cells/μL. Next, 30 μL of the PEG solution was loaded into a round-bottom, ultralow attachment 384-well plate (Corning). A 0.3 μL drop of the DEX phase containing 15 × 10^3^ cells was dispensed into each well using a robotic liquid handler (SRT Bravo) (Agilent Technologies). The plate was incubated at 37°C for 24 h to allow formation of a spheroid in each DEX phase drop within each microwell. Phase contrast images of spheroids were captured using an inverted fluorescence microscope (Axio Observer A1) (Zeiss).

### Embedding cancer cell spheroids in partitioned collagen

Aqueous DEX500k phase solution of 14% (w/v) was prepared in a complete growth medium and mixed with an equal volume of 4 mg/ml collagen solution to obtain a solution of 7% (w/v) DEX and 2 mg/ml collagen. After BT474 spheroids formed, 10 μL of the collagen-DEX solution was dispensed into each well containing the spheroids submerged in the PEG solution. These concentrations of PEG, DEX, and collagen were selected to replicate partition of 2 mg/ml collagen in a PEG-DEX system of 5% (w/v) PEG-7% (w/v) DEX system. The 384-well plate containing spheroids was maintained at 4°C for 30 min before dispensing the collagen-DEX solution. Again, the robotic liquid handler was used for uniform dispensing of the solution. The 384-well plate was kept on an ice tray for 60 min to allow collagen partitioning take place and another 30 min in room temperature. Then, the plate was incubated at 37°C to allow the collagen to gel. The 384-well plate was not transferred directly from 4°C to 37°C incubator to prevent potential thermal shock to cells.

### Statistical analysis

Data from the experiments were expressed as mean ± standard error. Two-way ANOVA with Bonferroni *post hoc* tests (MINITAB) were used to compare means among experimental groups. Each group had at least *n* = 4 replicates. Statistical significance was defined at *p* < 0.05.

## Results and discussion

### Characterization of ATPS

**Figure 1:**
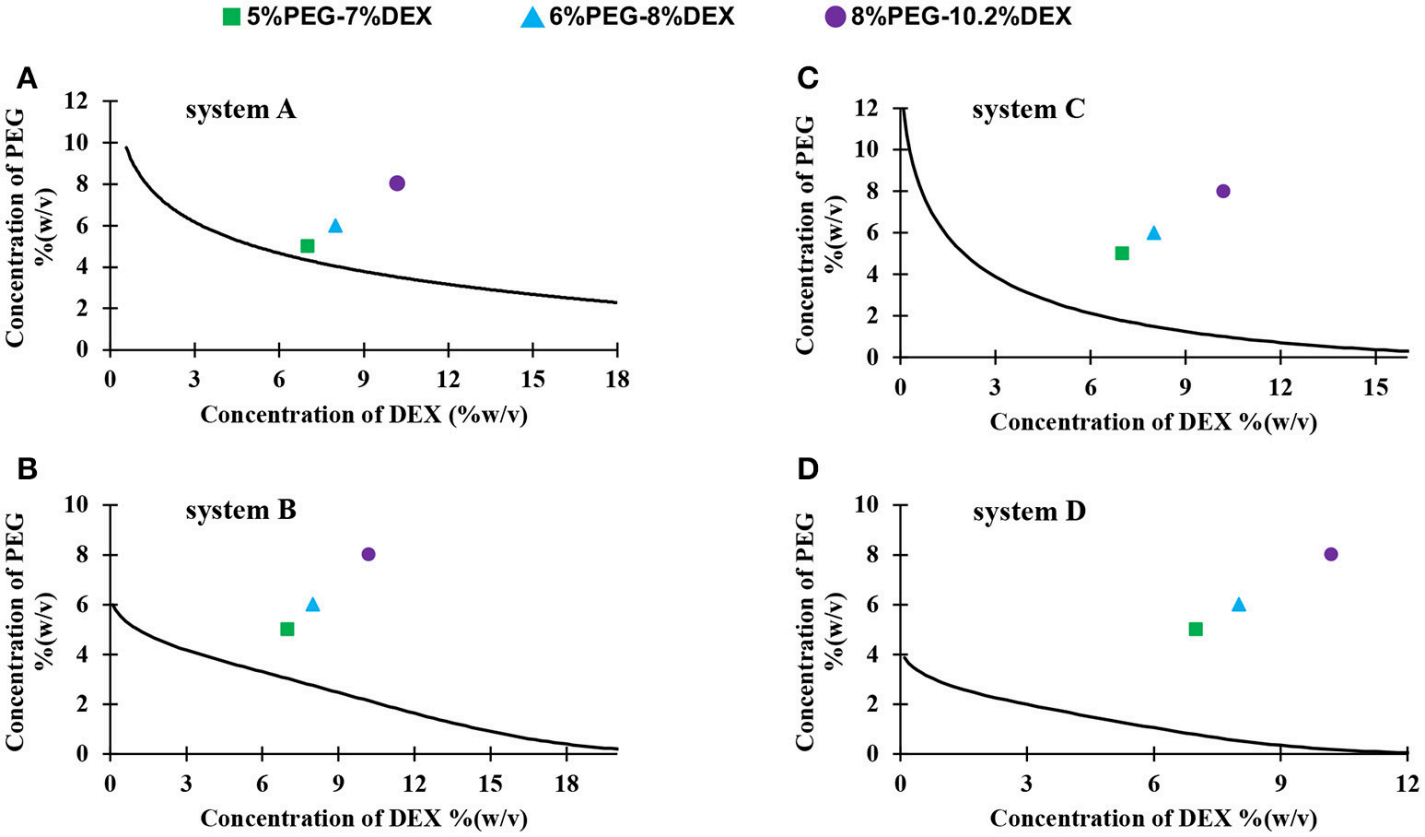
Binodal curves of four systems made with different molecular weights PEG and DEX: **(A)** PEG8k-DEX40k, **(B)** PEG8k-DEX500k, **(C)** PEG35k-DEX40k, and **(D)** PEG35k-DEX500k. Colored symbols show the resulting concentrations of PEG and DEX in the partition assay; square: 5%PEG-7%DEX, triangle: 6%PEG-8%DEX, and circle: 8%PEG-10.2%DEX.

We constructed binodal curves for four systems A-D made with different molecular weights of PEG and DEX using a titration method (Figure 1) (Atefi et al., 2016). Each curve represents critical concentrations of phase-forming polymers above which two distinct aqueous phases formed. Construction of binodal curves was necessary to determine working concentrations of the polymers to give two-phase systems. We selected the stock concentrations of PEG and DEX (15% PEG-21% DEX, 18% PEG-24% DEX, and 24% PEG-32% DEX) because two-phase solutions made at these concentrations contained equal volumes of PEG-rich top phase and DEX-rich bottom phase, making it convenient to measure volume of each phase during partition experiments. This also allowed visual comparison of partition of collagen in the bottom phase of two-phase systems made with the three sets of concentration pairs in each system A-D. As expected, the binodal curve was more asymmetric when the difference in molecular weights of PEG and DEX polymers increased (Atefi et al., 2016). System C had the most symmetric binodal curve, whereas system B had the most asymmetric binodal curve (Figure 1).

### Measurements of collagen concentration using hydroxyproline assay

**Figure 2:**
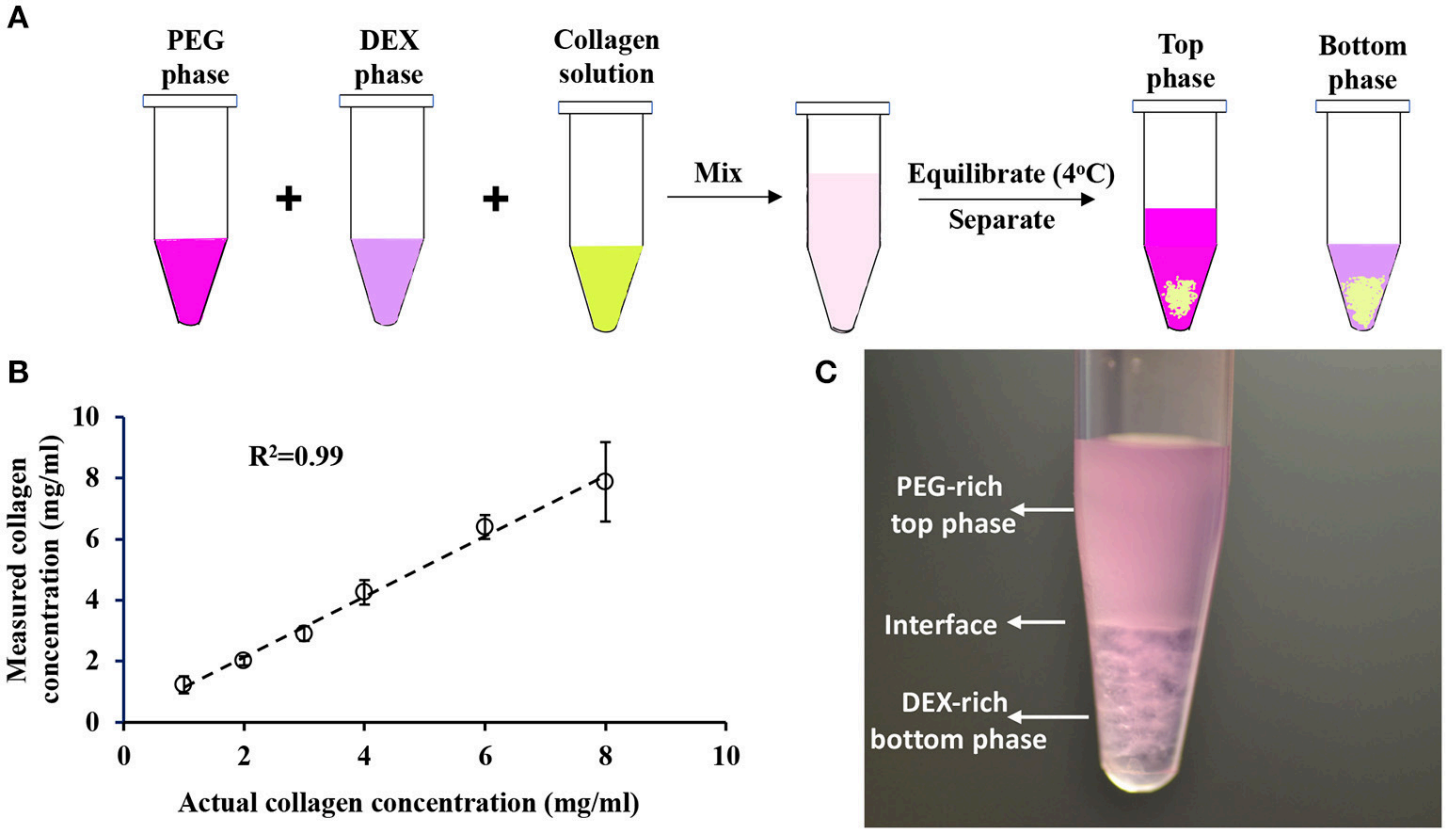
**(A)** Schematic of collagen partition experiment in ATPS. **(B)** Quantification of collagen concentration using hydroxyproline assay. Error bars represent standard deviations. **(C)** Collagen partition assay containing equal volumes of 15% PEG35k, 21% DEX500k, and 2 mg/ml collagen results in formation of 5% PEG-7% DEX ATPS.

We quantified collagen content in samples using a commercially available hydroxyproline assay kit. To assess the accuracy of the assay, we prepared known concentrations (1, 2, 3, 4, 6, and 8 mg/mL) of collagen standards in serum-free RPMI medium to avoid interference of serum proteins with the hydroxyproline amino acids present in the collagen standards. We acid hydrolyzed collagen standards for 16 h and determined the protein concentration. The result in Figure 2B shows a strong correlation between the actual collagen concentrations used in the experiment and the measured collagen concentrations, indicating that this assay can precisely predict collagen concentration in sample solutions. Importantly, we generated hydroxyproline standard curves using distilled water, and PEG and DEX solutions and showed that polymers do not cause interference in the absorbance signal from the samples when quantifying collagen concentration in an aqueous polymeric solution (Figure S1).

### Collagen partition in ATPS

We performed collagen partition experiments with systems A-D. We mixed equal volumes of PEG solution, DEX solution, and collagen solution in a microcentrifuge tube, equilibrated the mixture at 4°C, and transferred top and bottom phase into separate vials (Figure 2A). From each system, we selected three pairs of concentrations of aqueous phases: 15% PEG-21% DEX, 18% PEG-24% DEX, and 24% PEG-32% DEX. The resulting polymer concentrations in the collagen partition assay were 5% PEG-7% DEX, 6% PEG-8% DEX, and 8% PEG-10.2% DEX, respectively (Figure 1). These pairs located above the binodal curve and resulted in two-phase systems which we visually confirmed by observing a clear interface. We defined a partition coefficient (K_c_) as the ratio of collagen concentration in bottom phase and the total collagen concentration used in each assay, i.e.,

$$\begin{array}{l} {\text{K}}_{\text{c}}\;=\;\frac{Collagen\;concentration\;in\;bottom\;phase}{Total\;collagen\;concentration}\times 100\% \end{array}$$

Figure 2C shows an image from a collagen partition assay using the 5% PEG-7% DEX pair of system A. A distinct interface between the PEG-rich top phase and DEX-rich bottom phase is clear. This two-phase system gave a collagen partition coefficient of 61 ± 3%.

**Figure 3:**
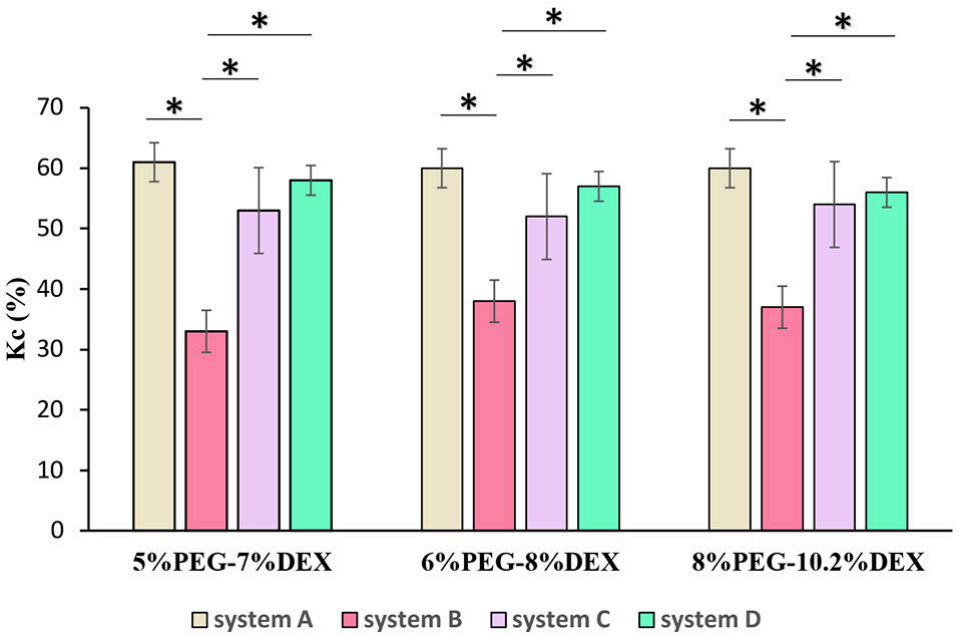
Collagen partition coefficient (K_c_) in four different systems using 5% PEG-7%DEX, 6% PEG-8% DEX, and 8% PEG-10.2% DEX concentration pairs. *n* = 4, *p < 0.05 (ANOVA with Bonferroni *post-hoc* test).

Figure 3 shows partition coefficients of collagen in systems A-D, using three concentration pairs from each system: 5% PEG-7% DEX, 6% PEG-8% DEX, and 8% PEG-10.2% DEX. In the 5% PEG-7% DEX pair, collagen partition coefficient was the highest in system A (61 ± 3%). When the molecular weight of DEX increased to 500 kDa but the molecular weight of PEG was kept constant (system B), the partition coefficient significantly decreased to 33 ± 4%. Increasing the molecular weight of PEG from 8 kDa in system B to 35 kDa in system D but keeping the molecular weight of DEX constant at 500 kDa significantly increased the partition coefficient to 58 ± 2%. With systems A-D, we obtained similar results using 6% PEG-8% DEX and 8% PEG-10.2% DEX pairs of concentrations.

These results suggest that collagen partition in ATPS is highly sensitive to molecular weight of phase polymers. That is, reducing molecular weight of a polymer increases the propensity of collagen to partition to the aqueous phase of that polymer. This finding is consistent with a previous report that proteins in non-ionic ATPS attract to the aqueous phase with smaller polymer molecules if all other conditions such as polymer concentration, temperature, and salt concentrations are kept constant (Albertsson et al., 1987). Another interesting finding contrary to the above conclusion emerged from our results. That is, increasing PEG molecular weight from 8 kDa in system A to 35 kDa in system C but keeping DEX molecular weight fixed at 40 kDa decreased the partition coefficient by 8%. However, when PEG molecular weight increased from 8 kDa in system B to 35 kDa in system D but DEX molecular weight was fixed at 500 kDa, the partition coefficient significantly increased again. These results suggest that the above rule about the effect of molecular weight of phase polymers on significant uneven partition of collagen between the two aqueous phases is valid only when there is a large difference between molecular weights of the polymers. Finally, comparing results among the three pairs of PEG and DEX in a specific system, i.e., 5% PEG-7% DEX, 6% PEG-8% DEX, and 8% PEG-10.2% DEX, showed no statistical difference in collagen partition coefficient (Figure 3).

### Collagen partitioning in ATPS for tumor tissue engineering

**Figure 4:**
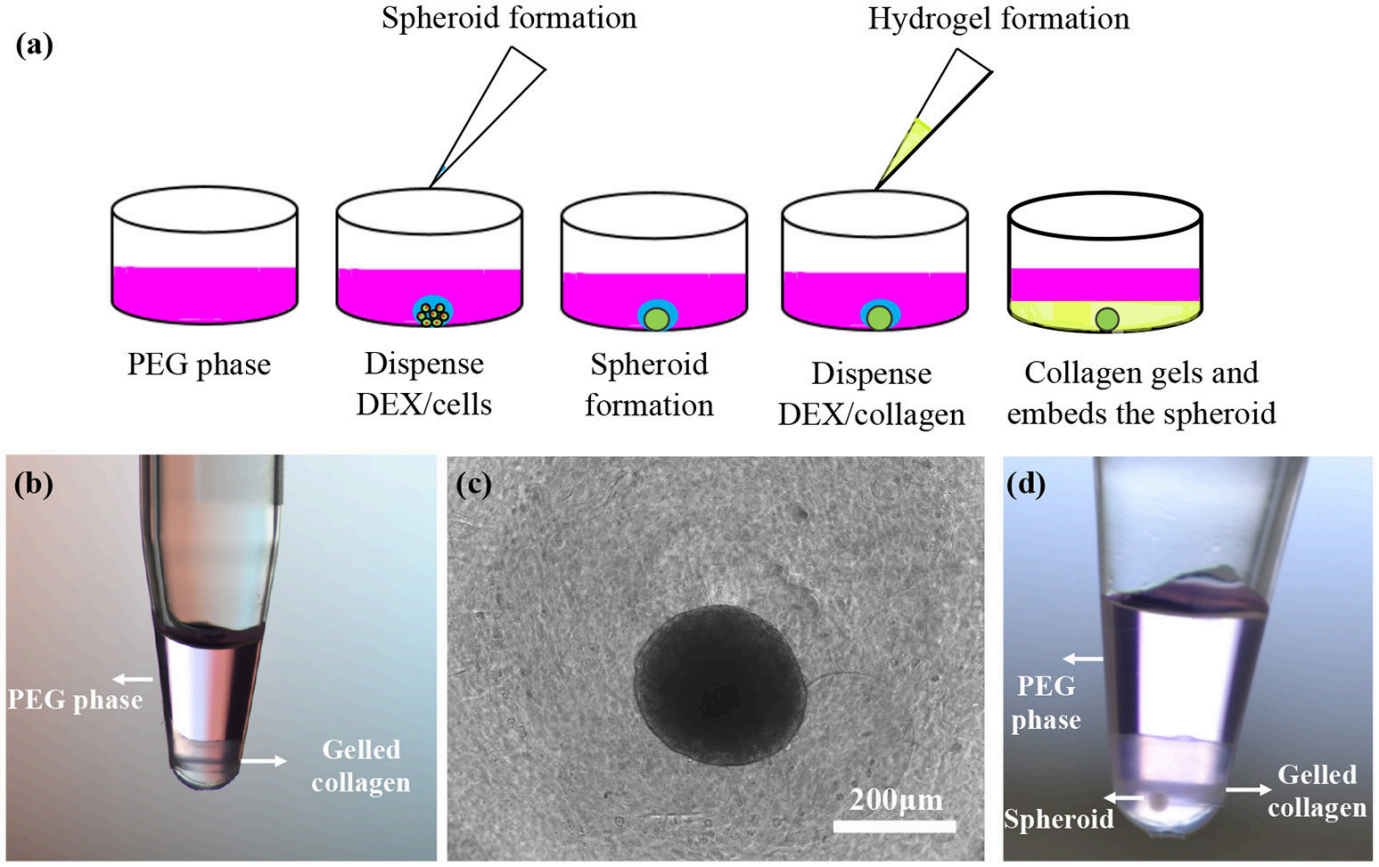
**(a)** Schematic representation of spheroid formation and embedding in a collagen gel using ATPS in two pipetting steps. **(b)** A solution of 5 μL 14% DEX 500k and 5 μL 4 mg/ml collagen solution was dispensed into 30 μL of a 6.6% PEG 35k solution. Collagen gelled in the bottom phase during incubation. **(c,d)** Top and side views of a BT474 spheroid embedded in a collagen gel partitioned to the DEX phase.

A previous study showed that collagen primarily partitions to the interface of ATPS (Moraes et al., 2013). This property was used to generate low-volume collagen microdrops (<10 μL) that mimicked matrix contraction in tissue environments. Here, we demonstrated the utility of collagen partitioning to the DEX phase of ATPS by creating a 3D microtissue. In our study, we quantitatively showed improved partition of collagen to DEX phase of several ATPS. We selected system D with 5% PEG-7% DEX because our preliminary experiments and previous work showed that the PEG35k-DEX500k ATPS (system D) gives consistently-sized and compact spheroids over wide range of polymer concentrations compared to other ATPS used (Ham et al., 2016a). From this ATPS, we used the 5% PEG-7% DEX pair because it gave a similar collagen partition coefficient to those from the 6% PEG-8% DEX and 8% PEG-10.2% DEX pairs (Figure 3) but at lower concentration of the polymers that is preferable for cellular applications with ATPS (Tavana et al., 2009; Atefi et al., 2014). After a spheroid formed in each well of a 384-well plate, we dispensed 10 μl of the DEX solution containing collagen to the wells containing spheroids on the well-bottom submerged in the PEG phase solution (Figure 4a). Because the DEX phase is denser than the PEG phase, it sank to the bottom of the wells. Due to the propensity of collagen toward the DEX phase, it remained in the DEX phase during incubation. We confirmed confinement of collagen to the DEX phase by dispensing equal amounts of the PEG and DEX phases and collagen in a PCR tube (Figure 4b). Incubating the plate at 37°C led to the gelation of collagen that surrounded the spheroid (Figure 4c). We visually confirmed that the spheroid was embedded in collagen by performing this assay in a PCR tube (Figure 4d). Additionally, we removed the gelled collagen from the wells and mounted them on a glass slide for imaging (not shown).

Collagen embedding of spheroids has been previously done using different approaches. For example, after forming spheroids using a rocking method, spheroids were transferred into a well plate and then overlaid with a collagen solution (Liang et al., 2011). Liver cell spheroids were formed using a hanging drop method and transferred into a well plate containing a collagen solution to produce 3D collagen gel cultures (Yip and Cho, 2013). These methods of forming spheroids have several limitations such as medium evaporation from the hanging drops and difficulty of handling the hanging drop culture plates, and inconsistently-sized spheroids made with the plate rocking technique (Lemmo et al., 2014). More importantly, spheroids have to manually be transferred to another plate and the medium has to be removed before the collagen solution is dispensed onto the spheroids. This approach is labor-intensive and risks losing the spheroid during aspiration of medium. Unlike these methods, our method eliminates the tedious steps of transferring spheroids to a new plate and medium aspiration. We used the same ATPS formulation both to prepare spheroids and to partition collagen to embed the spheroids. The entire process was done in two pipetting steps (Figure 4a): First, a DEX phase drop containing cancer cells was dispensed into the PEG phase to form a spheroid in the drop phase. Then, a collagen-containing DEX phase drop was dispensed to merge with the spheroid containing drop and form a hydrogel that entrapped the spheroid. This approach significantly simplified the preparation of microtissues. Additionally, because partitioning of proteins in ATPS is independent of polymer concentrations (Albertsson, 1970), this approach can conveniently produce collagen gels of desired concentrations to reproduce mechanical properties of tumors *in vivo* (Plodinec et al., 2012), matrix stiffness and porosity (Miron-Mendoza et al., 2010), and collagen permeability (Ramanujan et al., 2002). Our quantitative results of partition of collagen in ATPS (Figure 3) is key to facilitate this approach. This also allows us to produce hydrogels of different sizes and stiffness values simply by changing the volume of the DEX phase drops containing desired concentrations of collagen. This simplified approach is especially a major advantage for high throughput applications such as cancer drug screening (Shahi Thakuri et al., 2016). Encapsulating spheroids using collagen partitioning in ATPS is a novel technique to develop an *in vitro* 3D tumor model. More complex tumor models can also be conveniently developed by including other cellular components of tumor microenvironment such as fibroblasts and immune cells (Balkwill et al., 2012; Ham et al., 2016b, 2018).

## Conclusions

We used a quantitative approach to establish that collagen partitioning in polymeric ATPS is highly sensitive to polymer molecular weight. Using this property, we improved partitioning of collagen to the DEX phase of a PEG-DEX ATPS and employed this approach to conveniently develop physiologically-relevant *in vitro* 3D breast tumor models. This new technique will enable future studies to investigate the impact of components of tumor microenvironment on different functions of cancer cells.

## Author contributions

HT designed the project, helped write, and edited the manuscript. SS conducted experiments, analyzed data, and wrote the manuscript.

### Conflict of interest statement

The authors declare that the research was conducted in the absence of any commercial or financial relationships that could be construed as a potential conflict of interest.
