# Supplementary material for: Collagen Partition in Polymeric Aqueous Two-Phase Systems for Tissue Engineering
Source: Front Chem. 2018 Sep 4;6:379. doi: 10.3389/fchem.2018.00379 (PMC6132203; doi:10.3389/fchem.2018.00379)
Supplement: Supplementary file 1 [file Presentation_1.pdf]

Supplementary Information

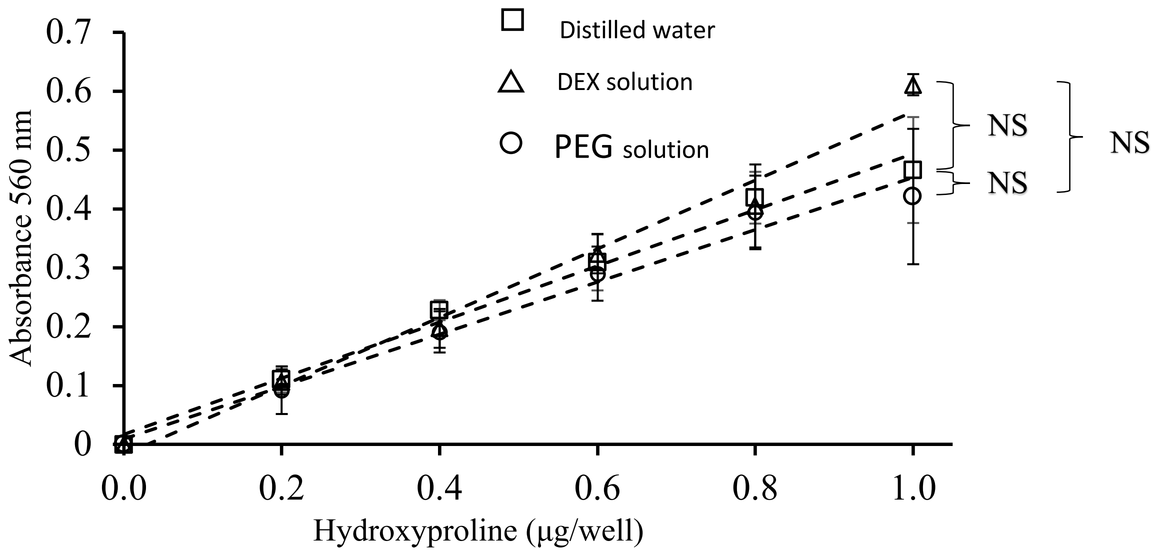

Figure S1. Standard curve for hydroxyproline standards prepared in distill water, 10% (w/v) DEX500k solution, and 10% (w/v) PEG35k solution. All samples were hydrolyzed and absorbance was measured following the manufacturer's protocol. Statistical method: two-way ANOVA. NS: statistically nonsignificant.
